# Supplementary material for: Antibody and T Cell Responses against SARS-CoV-2 Elicited by the Third Dose of BBIBP-CorV (Sinopharm) and BNT162b2 (Pfizer-BioNTech) Vaccines Using a Homologous or Heterologous Booster Vaccination Strategy
Source: Vaccines (Basel). 2022 Mar 30;10(4):539. doi: 10.3390/vaccines10040539 (PMC9025723; doi:10.3390/vaccines10040539)
Supplement: Supplementary file 1 [file vaccines-10-00539-s001.zip › vaccines-1632887-supplementary.pdf]

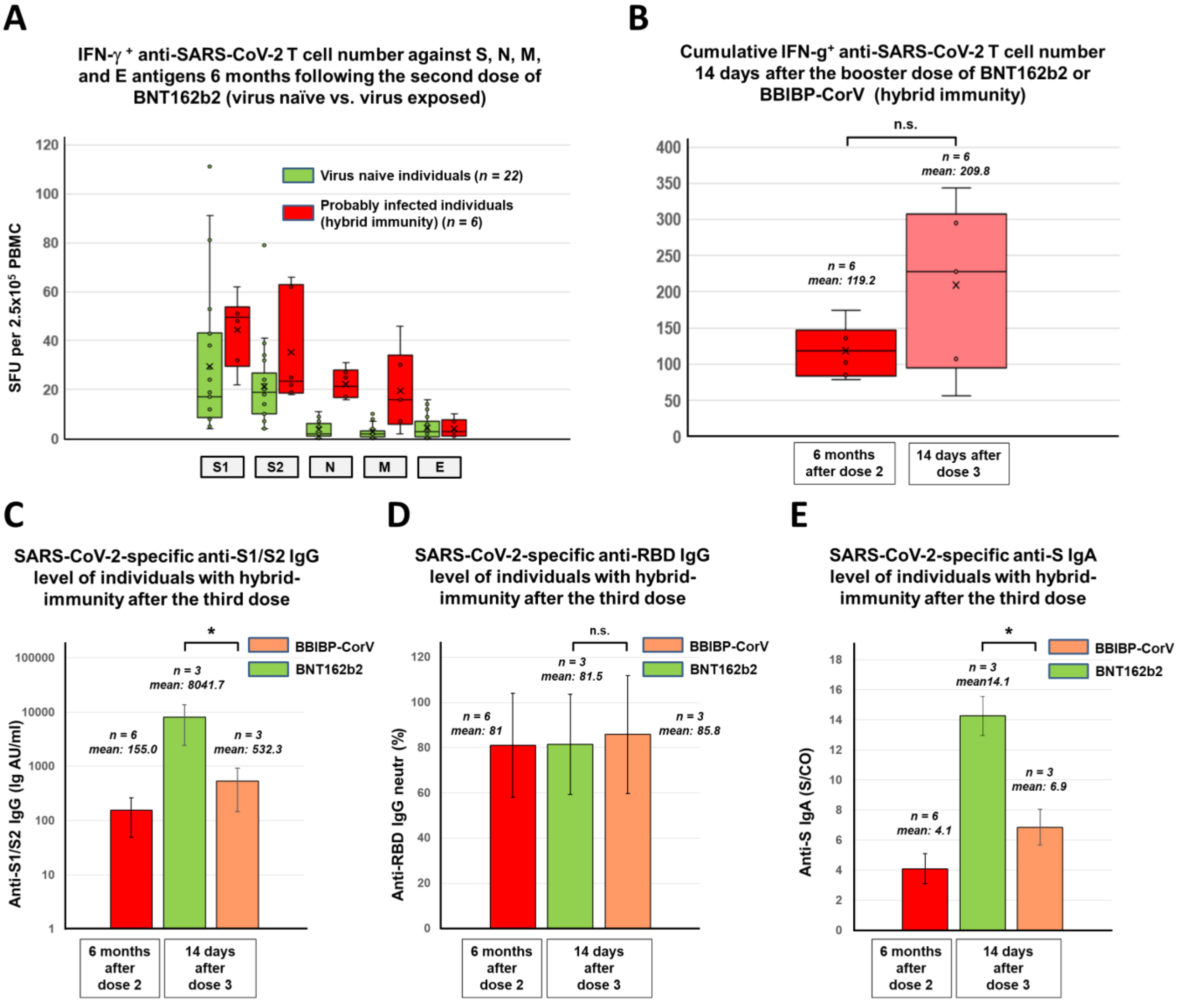

Figure S1. SARS-CoV-2-specific antibody and T cell responses of individuals with hybrid-immunity before and after the third dose of mRNA (BNT162b2) or inactivated virus (BBIBP-CorV) vaccine. All participants were immunized with a two-dose BNT162b2 regimen and then they were probably infected with SARS-CoV-2 according to their anti-N and anti-M T cell responses. The cumulative IFNγ-positive T cell responses (total SFU against S1, S2, N, M, and E antigens) (B) or the detailed T cell responses against each antigen (A) were evaluated by the T-SPOT Discovery SARS-CoV-2 ELISpot assay for 6 virus-experienced participants 6 months after receiving the second dose of the BNT162b2 vaccine (A) and 14 days after receiving the booster dose of the BNT162b2 or BBIBP-CorV vaccine (B). The SARS-CoV-2-specific anti-S1/S2 IgG levels (C), the anti-RBD IgG levels (D), and the anti-S IgA levels (E) were determined by the LIAISON SARS-CoV-2 S1/S2 IgG test, the SARS-CoV-2 surrogate virus neutralization test (sVNT), and the SARS-2 Covid S IgA assay, respectively, for 6 virus-experienced participants 6 months after receiving two doses of the BNT162b2 vaccine and 14 days after receiving the booster dose of the BNT162b2 (n=3) or BBIBP-CorV (n=3) vaccine. Statistical analyses were performed applying the Wilcoxon signed rank test or Student's t-test as appropriate, and p values of <0.05 were considered statistically significant (*) while p-values of >0.05 were considered non-significant (n.s.).
